# Awareness of malaria and treatment-seeking behaviour among persons with acute undifferentiated fever in the endemic regions of Myanmar

**DOI:** 10.1186/s41182-017-0070-9

**Published:** 2017-12-04

**Authors:** Phyo Aung Naing, Thae Maung Maung, Jaya Prasad Tripathy, Tin Oo, Khin Thet Wai, Aung Thi

**Affiliations:** 1 Department of Medical Research, Ministry of Health and Sports, No. 5, Ziwaka Road Dagon Township, Yangon, 11191 Myanmar; 2 International Union Against Tuberculosis and Lung Disease, The Union South-East Asia Regional Office, New Delhi, India; 3 National Malaria Control Program, Ministry of Health and Sports, Naypyitaw, Myanmar

**Keywords:** Treatment-seeking behaviour, Myanmar, Malaria/diagnosis, Health knowledge, Attitudes, Practice, Plasmodium infections

## Abstract

**Background:**

Myanmar has a high burden of malaria with two-third of the population at risk of malaria. One of the basic elements of the Roll Back Malaria Initiative to fight against malaria is early diagnosis and treatment within 24 h of fever. Public awareness about malaria is a key factor in malaria prevention and control and in improving treatment-seeking behaviour.

**Methods:**

A large community-based survey was carried out in 27 townships of malaria endemic regions in Myanmar in 2015 which reported on the knowledge, behaviour and practices around malaria in the general population. We used the data already collected in this survey to assess (i) general public awareness of malaria and (ii) treatment-seeking behaviour and associated factors among persons with acute undifferentiated fever.

**Results:**

A total of 6597 respondents from 6625 households were interviewed (response rate of 99.5%). About 85% of the respondents were aware that mosquito bite was the mode of transmission of malaria and 90% mentioned that malaria was preventable. However, only 16% of the respondents knew about anti-malaria drug resistance. There were certain misconceptions about the transmission of malaria such as dirty water, same blood group, sharing shelter, sleeping/eating together and poor hygiene. Health facility staff were the most common source of information about malaria (80%). Nearly one-fourth (23%) of the respondents with fever resorted to self-medication. Around 28% of the respondents with fever underwent blood testing, less than half of whom (44%) were tested within 24 h. Elderly age group, females, those with poor knowledge about malaria and those residing in non-Regional Artemisinin Resistance Initiative townships were associated with poor treatment-seeking behaviour in case of fever.

**Conclusion:**

Although there is fair knowledge on mosquito bite as a mode of transmission and prevention of malaria, there are some misconceptions about transmission of malaria. Those having poor knowledge about malaria have poor treatment-seeking behaviour. A considerable number of respondents seek care from informal care providers and seek care late. Thus, there is a need to promote awareness about the role of early diagnosis and appropriate treatment and address misconceptions about transmission of malaria.

## Background

Malaria is an acute febrile illness caused by *Plasmodium* parasite. It is considered a serious public health threat because of its severity and often fatal outcome. Over half of the population is known to be at risk of malaria globally; and most of them are children under-5 years of age [1]. World Health Organization (WHO) reports that in the Asia and Pacific region, which covers 22 countries, about 2.1 billion people (80% of the total population) are at risk of getting malaria. A total of 212 million cases and an estimated 429,000 deaths were reported in 2015 globally [1].

Myanmar has a high burden of malaria with more than two-third of the population at risk of malaria. Myanmar has the greatest malaria incidence in the Greater Mekong Sub-region (GMS) with 253 cases per 100,000 population [2]. A total of 183,000 confirmed cases were reported in the country in 2015 against an estimated number of 240,000 [3]. Myanmar, located in the tropical zone, provides a favourable wet and moist climate for breeding of *Anopheles* mosquito. Nearly three-fourth of the cases of malaria are caused by *Plasmodium falciparum*. Myanmar is at the third position among the countries in the South East Asian Region (SEAR) with contribution of malaria cases, and at top among the countries in GMS which is known for artemisinin resistance [4].WHO has emphasised early diagnosis and prompt treatment within 24 h of onset of symptoms to decrease the risk of severe complications and onward transmission [5]. One of the basic elements of the Roll Back Malaria Initiative to fight against malaria is early diagnosis and treatment of malaria (EDTM). It is thus recommended that patients should seek early medical advice following the onset of fever, a common symptom of malaria [5].

Studies in the SEAR such as Bangladesh, India, Nepal and Bhutan have found that public awareness about malaria is a key factor in malaria prevention and control. It also plays a key role in improving treatment-seeking behaviour [6–9]. Earlier studies in this region have reported that poor treatment-seeking behaviour was associated with poor socio-economic status, rural residence, proximity to health facilities, accessibility to trained providers, availability of transportation and knowledge of malaria [10–13]. However the studies in Myanmar were done in a selected sample and thus did not represent the whole country [11, 12, 14, 15].

A large community-based survey was carried out in the endemic regions of Myanmar in 2015 which reported on the knowledge, behaviour and practices around malaria in the general population. We used the data already collected in this survey to assess (i) general public awareness of malaria and source of information about malaria and (ii) treatment-seeking behaviour and associated factors among persons with acute undifferentiated fever in the previous 2 weeks. This information would be useful in designing appropriate health education content and in formulating effective interventions to improve health-seeking behaviour in the event of fever in the endemic regions.

## Methods

### Study design

It was a cross-sectional analytical study involving analysis of secondary data from a community-based survey conducted by the National Malaria Control Program (NMCP) in Myanmar in 2015. The key outcome variables are knowledge about cause, transmission and prevention of malaria and drug resistance. Other outcome variables are the proportion of respondents with undifferentiated fever who had poor treatment-seeking behaviour, got their blood tested for malaria and timing of test done.

### General setting

The Republic of the Union of Myanmar is one of the South East Asian countries neighboured by countries like Bangladesh, India, China, Laos and Thailand in the North and East [16]. It is divided administratively, into the capital territory (Nay Pyi Taw Council Territory), seven states and seven regions. There are 74 districts with 330 townships. According to the last census, Myanmar has an estimated population of 51.5 million with nearly 70% residing in rural areas [17]. Ministry of Health and Sports (MOHS) provides health care services to the whole population through hospitals by Department of Medical Services (DMS). Primary health care services are provided by the Department of Public Health especially in the rural areas [3].

### Specific setting: epidemiological profile, intervention policies and strategies for malaria in Myanmar

Malaria is endemic in 284 out of 330 townships in Myanmar. The country constitutes of high transmission (> 1 case per 1000 population), low transmission (0–1 case per 1000 population) and malaria-free areas (zero cases) represented by 16, 44 and 40% of the total population, respectively [2]. It remains a public health problem due to climatic and ecological changes, population migration and ecological development activities such as mining, forestry and development of drug resistant *Plasmodium falciparum* parasite.

In Myanmar, National Malaria Control Programme (NMCP) is carrying out malaria control activities in line with the Global and National Malaria Control Strategies in collaboration with national and international partners. The key programme interventions include information, education and communication; free distribution of insecticide-treated nets in areas of high malaria transmission; indoor residual spraying; diagnostic testing for suspected malaria cases; and artemisinin-based combination therapy (ACT), all of which are provided free-of-cost. According to the anti-malarial treatment policy, case management with ACT was initiated in all endemic townships in 2009 [18].

### Community-based survey

**Fig. 1:**
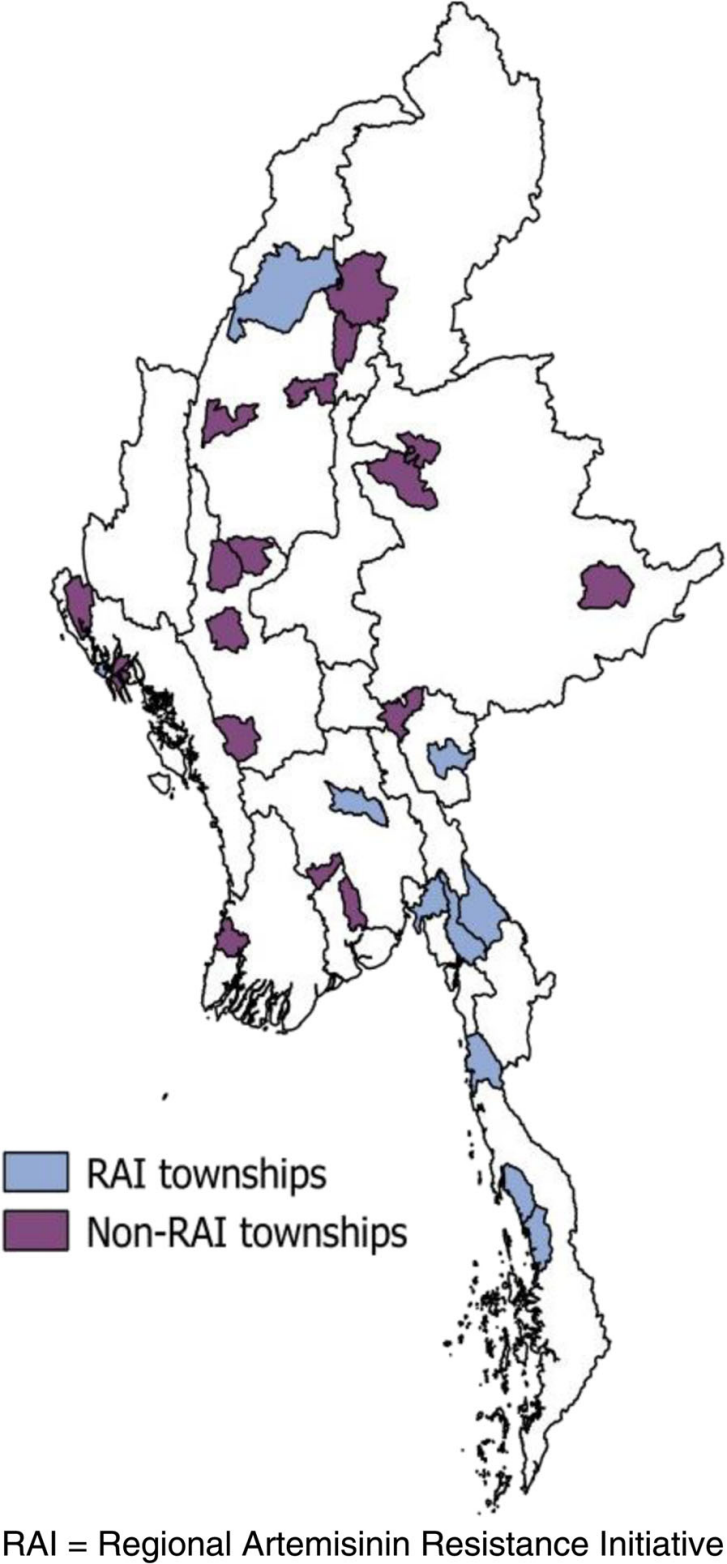
Map showing the malaria endemic townships where the community based survey was carried out in Myanmar in 2015

A nationwide community-based survey was conducted jointly by the Department of Medical Research (DMR) and NMCP in 2015 to understand the knowledge, attitude and health-seeking behaviour towards malaria in the general population in all endemic States and Regions of Myanmar except Mandalay Region and Chin State. A multistage sampling procedure was used in this survey. Firstly, 27 townships were selected using the probability proportionate to size (PPS) method. At the township level, 8 villages were randomly selected from each township. In each village, 25–30 households were systematically selected by using a predefined list of village households. Figure 1 shows a map depicting the malaria endemic townships where the community-based survey was carried out. In the selected households, face to face interview was conducted with preferably the female adult respondent or any other adult using a semi-structured questionnaire by trained interviewers. Questionnaires were pre-tested and all interviewers were well trained in each State/Region by NMCP and DMR.

A total of 6625 households in 216 villages located in 27 townships were covered out of which 6597 respondents completed the interview. Survey data was double entered and validated using EpiData Entry software (version 3.1, EpiData Association, Odense, Denmark). This community-based malaria survey database is available with the NMCP, Ministry of Health and Sports, Myanmar.

### Study population

For the first objective, the study population comprises of the general population in endemic regions of Myanmar covered under the survey. Persons with acute undifferentiated fever in the last 2 weeks in endemic regions of Myanmar constitute the study population for the second objective.

### Operational definition

Poor treatment-seeking behaviour is defined as no medication or self-medication or seeking treatment from traditional healer.

#### Knowledge score

Questions related to knowledge about malaria—its transmission, prevention, anti-malarial drug resistance and management were scored as 1 (yes) and 0 (no). A total of 14 questions on the abovementioned questions were included in the scoring with a Cronbach’s score of 0.76 (measure of internal reliability). The maximum and minimum scores were 0 and 14. The median (IQR) of the scores was 7 (3–10). The scores were then added up to get a knowledge score for each individual in the survey. The score was then categorised into high and low based on the median cut-off value.

#### Regional Artemisinin Resistance Initiative (RAI) townships

In 2009–2010, Myanmar reported suspected artemisinin resistance. Myanmar Artemisinin Resistance Containment (MARC) Framework was developed and endorsed in April 2011 to control the emergence of artemisinin resistance. Areas of artemisinin resistance are stratified into three Tiers**.** *Tier 1* are areas where there is credible evidence of artemisinin resistance; *tier 2* includes areas with significant inflow of people from tier 1, including those immediately bordering Tier 1; *tier 3* are areas with no evidence of artemisinin resistance and limited contact with Tier 1 areas. There are 31 townships in tier 1, 21 in tier 2 and 258 in tier 3. In 2013, the project was transferred to the Three Millennium Development Goal (3MDG) and renamed as Regional Artemisinin Initiative (RAI) in March, 2014. The RAI project area includes 52 townships in tiers 1 and 2. In 2015, the RAI area was expanded to 72 townships, and in 2016, the RAI area was further extended up to 76 townships [19]

### Data analysis

Data were extracted from EpiData database and imported into STATA (version 11, StataCorp, TX, USA) for analysis. Measures of descriptive statistics, i.e. proportions were used to summarise categorical variables related to knowledge about malaria-its transmission, prevention, drug resistance and management and source of information. These are presented in the form of bar diagrams. Incidence of fever was reported by different socio-demographic characteristics in the form of proportions. Chi-square test was used to study the association between socio-demographic variables and poor treatment-seeking behaviour. Multivariable logistic analysis (enter method) was performed to find out the factors associated with poor treatment-seeking behaviour in the event of fever. Due to the clustered sample design and non-response in the survey, weights were calculated where each participant was given a value/weight. Sampling and non-response weights were used to calculate the final weight which is a product of the two weights. This study reports weighted estimates.

## Results

### Community awareness about malaria

Out of 6625 households, 6597 respondents, i.e. one respondent from each household (response rate of 99.5%), completed the interview. There were 31,165 members in these households.

**Fig. 2:**
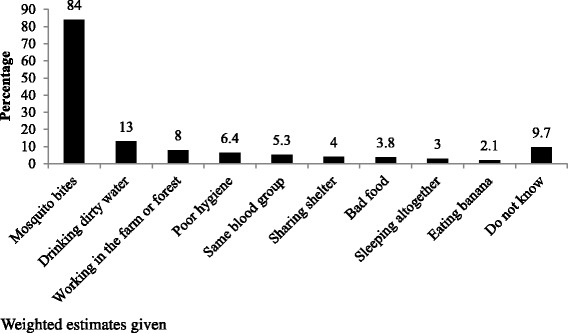
Awareness about cause and transmission of malaria among the general population in the endemic regions of Myanmar in 2015

**Fig. 3:**
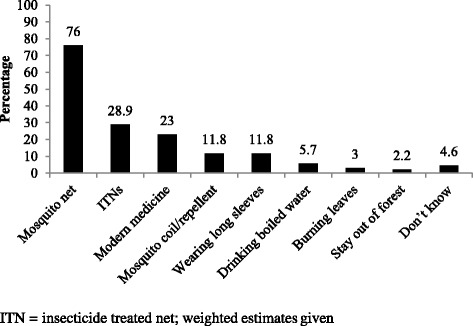
Awareness about prevention of malaria among the general population in the endemic regions of Myanmar in 2015

Of all the household respondents interviewed, 5771 (87.5%) had heard of malaria, among whom 5004 (87%) knew mosquito bites as the mode of transmission of malaria. Besides, other common misconceptions around transmission of malaria included drinking dirty water (13%), poor hygiene (6.4%), same blood group (5.3%), sharing shelter (4%), bad food (3.8%) and sleeping together (3%). Figure 2 Nearly 90% of respondents were of the view that malaria is preventable. The common methods of preventing malaria as reported by the respondents were mosquito nets (76%) followed by ITNs (28.9%) Fig. 3.

**Fig. 4:**
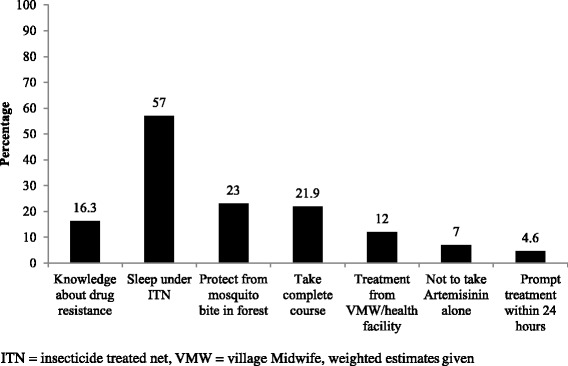
Awareness about anti-malarial drug resistance and its prevention among the general population in the endemic regions of Myanmar in 2015

**Fig. 5:**
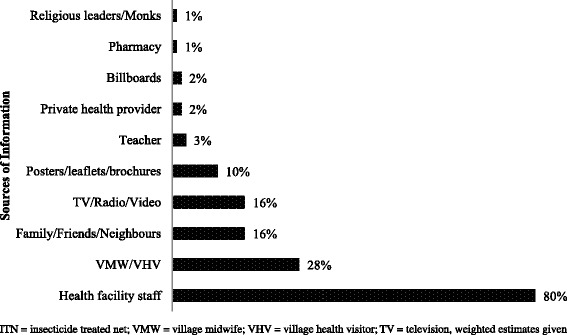
Source of information for knowledge about malaria among the general population in the endemic regions of Myanmar in 2015

Again, 90% of the respondents said that malaria can be treated, and among whom, 85% believed that modern medicine could treat malaria. Around 16.3% of the respondents were aware of anti-malarial drug resistance. The common methods for preventing drug resistance as reported are sleeping under ITN (49%) and protection from mosquito bite in the forest (21%) (Fig. 4). Figure 5 shows that health facility staff (80%) was the most common source of information about malaria followed by Village Health Volunteer (VHV)/Village MidWife (VMW) (28%) and family/friends/neighbours (16%).

### Treatment-seeking behaviour for acute undifferentiated fever

Of 6597 households, 938 (14.2%) households had any family member contracting fever within the last 2 weeks. Among 31,165 household members contacted, 1180 (3.8%) had fever within the last 2 weeks. The incidence of fever was significantly higher among children < 5 years (*p* < 0.001, 9.4%), certain occupation groups and manual labourers (4.8–5.0%) and those who had high knowledge scores (*p* = 0.04, 4.8%). Table 1.

**Table 1:** Socio-demographic characteristics of the respondents who reported fever within 2 weeks in the endemic regions of Myanmar, 2015

Characteristics		Total	Reported fever within 2 weeks *n* (%, 95% CI)	*p* value
Age group				*p* < 0.001
	Under 5	2673	250 (9.4, 7.2–12.0)	
	5–14	6638	337 (5.1, 3.3–7.3)	
	15–59	18,989	510 (2.7, 1.3–5.4)	
	60 and above	2793	83 (3.0, 1.2–4.8)	
Sex				*p* = 0.09
	Male	15,069	599 (4.0, 3.0–6.1)	
	Female	16,096	581 (3.6, 2.4–5.0)	
Occupation				*p* < 0.001
	Agriculture	18,950	625(3.4, 2.0–5.0)	
	Seller	2316	73(3.2, 1.4–5.5)	
	Casual labour	6719	325(4.8, 2.9–7.0)	
	Others	2785	138(5.0, 3.0–6.9)	
Education				*p* = 0.088
	Illiterate	4429	182(4.1, 3.0–5.0)	
	Up to primary	17,234	660(3.8, 2.3–5.5)	
	High school	8191	310(4.0, 2.8–5.4)	
	Higher education	805	18(2.2, 1.6–3.0)	
Townships				*p* = 0.18
	RAI	11,775	471(4.0, 3.1–5.0)	
	Non-RAI Townships	18,995	702(3.7, 2.8–4.8)	
Knowledge about malaria				p = 0.04
	High	1432	69(4.8, 3.0–6.8)	
	Low	29,733	1111(3.7, 2.6–5.0)	

Weighted estimates given

*RAI* Regional Artemisinin Resistance Initiative

Among persons with undifferentiated fever, only 28.2% underwent a blood test for confirmation of diagnosis. Less than half (~ 44%) of the blood tests were done within 24 h of fever. Nearly half of the respondents with fever approached a government health facility (48.4%) for treatment followed by self-medication (22.8%) and private clinic (22.1%) Table 2.

**Table 2:** Source of treatment and timing of blood test by age group among persons with undifferentiated fever within 2 weeks in the endemic regions of Myanmar in 2015

Age group (in years)		Under 5 (*n* = 246)	5–15 (*n* = 336)	15–59 (*n* = 504)	60 and above (*n* = 81)	Total (*n* = 1167)
Source of treatment		*n* (%)	*n* (%)	*n* (%)	*n* (%)	*n* (%)
	No medication taken	3 (1.2)	11 (3.3)	9 (1.8)	3 (3.7)	26 (2.2)
	Self-medication	42 (17.1)	78 (23.2)	128 (25.4)	18 (22.2)	266 (22.8)
	Traditional healer	8 (3.3)	10 (3.0)	17 (3.4)	5 (6.2)	40 (3.4)
	Government’s Health Centre	135(54.9)	155(46.2)	240(47.7)	34(41.9)	564(48.4)
	Private Health Centre	58(23.5)	78(23.2)	102(20.2)	19(23.5)	257(22.1)
	Others/DNK	0 (0)	4 (1.2)	8 (1.6)	2(2.5)	14 (1.2)
Blood test done		55 (22.5)	88 (26.2)	162 (32.1)	20 (24.7)	325 (27.8)
Timing of blood test		(*N* = 55)	(*N* = 88)	(*N* = 162)	(*N* = 20)	(*N* = 325)
	Within 24 h	26 (47.3)	35 (39.8)	75 (46.3)	8 (40.0)	144 (44.3)
	24–48 h	19 (34.6)	29 (33.0)	49 (30.3)	7 (35.0)	104 (32.0)
	More than 48 h	7 (12.7)	21 (23.9)	25 (15.4)	3 (15.0)	56 (17.2)
	Not sure	3 (5.5)	3 (3.4)	13 (8.0)	2 (10.0)	21 (6.5)

Weighted estimates given

*DNK* do not know

Elderly age group 60 years and above (9.0, 95% CI = 6.0–17.2, *p* = 0.01), female sex (1.5, 95% CI = 1.0–1.9, *p* = 0.03), illiteracy (3.5, 95% CI = 2.0–7.8, *p* = 0.0018), poor knowledge about malaria (2.9, 95% CI = 1.6–6.2, *p* < 0.001) and non-RAI townships (2.0, 95% CI = 1.4–2.6, *p* < 0.001) are associated with poor treatment behaviour in case of fever Table 3.

**Table 3:** Association of socio-demographic characteristics of respondents with poor treatment-seeking behaviour of those who reported fever within the last 2 weeks in the endemic regions of Myanmar, 2015

Characteristics		Total *N*	Poor treatment seeking behaviour (*N*)	Crude OR (95% CI)	*p* value	Adjusted OR (95% CI)	*p* value
Age group					0.008		
	Under 5	246	53	1.0		1.0	
	5–14	334	99	1.5 (1.1–2.3)		1.3 (0.8–2.5)	0.3
	15–59	500	154	1.6 (1.1–2.3)		1.4 (0.8–2.3)	0.2
	60 and above	81	64	10.7 (7.4–19.4)		9.0 (6.0–7.2)	0.01
Sex					0.008		
	Male	594	152	1.0		1.0	
	Female	567	181	1.4 (1.1–1-8)		1.5 (1.0–1.9)	0.03
Occupation					0.9		
	Agriculture	625	178	1.0		–	
	Seller	73	21	1.0 (0.6–1.7)		–	
	Casual labourer	319	94	1.1 (0.8–1.4)		–	
	Others	137	38	0.9 (0.6–1.5)		–	
Education level					< 0.001		
	Illiterate	177	88	3.4 (1.8–8.7)		3.3 (1.8–7.9)	0.0018
	Up to primary	656	172	1.2 (0.4–3.8)		1.0 (0.5–3.2)	0.3
	Middle and High school	307	68	1.0 (0.3–3.1)		0.8 (0.3–2.9)	0.5
	Higher education	18	04	1.0		1.0	
Knowledge score about malaria							
					< 0.001		
	Low	1092	329	3.8 (1.7–8.4)		2.9 (1.6–6.2)	<0.001
	High	69	07	1.0		1.0	
Type of townships							
	RAI townships	466	102	1.0	< 0.001	1.0	
	Non-RAI townships	695	231	1.8 (1.4–2.3)		2.0 (1.4–2.6)	<0.001

*OR* odds ratio, *RAI* Regional Artemisinin Resistance Initiative

Poor treatment-seeking behaviour is defined as no medication or self-medication or seeking treatment from traditional healer; weighted estimates given

## Discussion

The key highlights of the study are (a) although there is fair knowledge about transmission and prevention of malaria, there is poor awareness about anti-malarial drug resistance and certain misconceptions about the transmission of malaria, (b) health facility staff and VHV/VMW are the most common source of information about malaria, (c) nearly one-fourth of the respondents with fever resorted to self-medication, (d) less than one-third of the respondents with fever underwent blood testing; less than half of whom were tested within 24 h, and (e) elderly, females, those with poor knowledge about malaria and those residing in non-RAI townships were associated with poor treatment-seeking behaviour in case of fever.

Overall, there was fair knowledge about mosquito bites as a mode of transmission and prevention of malaria. The majority of the respondents reported that malaria could be transmitted from person-to-person through mosquito bites and could be prevented by the use of mosquito net. On the other hand, many misconceptions emerged around transmission of malaria, such as drinking dirty water, same blood group, sharing shelter, sleeping/eating together and poor hygiene. There was poor knowledge about drug resistance, its cause and prevention. These findings were consistent with other studies from Myanmar [20, 21]. The participants related malaria to water, food, hygiene and proximity which indicates poor awareness in the community. Although many people knew about the correct mode of transmission of malaria, i.e. mosquito bite, people who knew that also had some misconceptions as they cited multiple modes of transmission. Thus, a clear simple message that mosquito bite is the only mode of transmission of malaria should be disseminated. Also, health facility staff and others such as VMW/VHV, being the most common source of information about malaria, should be mobilised for disseminating the health education content. Other channels of dissemination such as television/radio/print media/village meetings should also be utilised in health education campaigns depending on the local context. Health education campaign should be designed to address the knowledge gaps identified in this study such as anti-malarial drug resistance, prevention of drug resistance through early diagnosis and treatment and misconceptions around the transmission of malaria.

Anti-malarial drug resistance is a major threat in this region with the emergence of artemisinin drug resistance. Prevention of drug resistance requires improving the way drugs are used through rational prescribing, better follow-up practices and patient compliance and discouraging self-medication or medication from informal providers [22]. The present study showed poor public awareness about drug resistance and its prevention and also reported substantial proportion of self-medication. Improving general awareness about drug resistance and its prevention might lead to improved compliance to therapy [23]. Thus, health education campaigns must be tailored to plug the gaps around causes and prevention of drug resistance in order to prevent the emergence and spread of resistance.

Nearly one-fourth of the respondents with fever resorted to self-medication. Available published literature shows that self-medication and seeking assistance from drug vendors were the most common practices in certain regions of Myanmar [12]. With 80% target of providing appropriate treatment within 24 h, WHO advocates strategies to improve home-based management of malaria, with drug retailers being seen as one possible channel [24, 25]. This could be one of strategies addressing timely and appropriate treatment of malaria in Myanmar.

A majority of respondents did not seek appropriate care within 24 h of fever. Delays of more than 24 h in care seeking has also been reported in other settings as well [12, 26]. More efforts are needed to ensure prompt malaria diagnosis and treatment since case fatality rises if treatment is delayed beyond 24 h after the onset of clinical symptoms [1]. The study reports that among those with undifferentiated fever, less than one-third get their blood tests done for malaria despite the fact that most of them seek care. This calls for intervention at the level of the health care provider including the drug retailers in terms of educating them on the need for testing for malaria in any case of fever before starting anti-malarial medications. Those who get their blood tested for malaria, less than half get it done within 24 h which requires a wider engagement of the community and rapid access to RDTs [27].

The study showed that families were more likely to seek appropriate care if patients were male or children under 15 years old similar to another cross-sectional study in Myanmar [12]. Women are dependent on family members for seeking care and thus, usually start with home remedies at the onset of symptoms which partly explains their poor treatment-seeking behaviour. On the other hand, there is more emphasis on children in a family and they are likely to be assisted by a caregiver in seeking treatment. Thus, programme interventions to improve treatment-seeking behaviour should prioritise these vulnerable groups.

It was also found that those who had greater knowledge score about malaria were over two times more likely to seek care from trained providers than those without sufficient knowledge which is supported by other studies in the literature [11, 28]. This also supports another finding in this study which reports significantly higher incidence of fever in respondents who had a higher knowledge score. This might be due to the fact that awareness about the condition leads to timely and appropriate decision making about seeking care. Future efforts should focus on improving knowledge for malaria treatment and prevention, particularly in endemic regions.

### Strengths and limitations

The major strengths of the study were that the data were obtained from a large national representative survey covering all the endemic regions of Myanmar; the response rate was high; the interviewers were well trained and supervised; and the subject is an identified national operational research priority. Data quality was ensured though double data entry and validation using EpiData entry software. We also adhered to The Strengthening the Reporting of Observational Studies in Epidemiology (STROBE) guidelines for the reporting of observational studies [29].

The study had some limitations as well. The study could not provide detailed information on early diagnosis and appropriate treatment (EDAT) pathway. The study also did not explore reasons for not seeking appropriate treatment in case of fever. Further qualitative studies are required to understand the reasons for the same.

## Conclusion

To conclude, although there is fair knowledge on transmission and prevention of malaria, there are some misconceptions about the transmission of malaria. Those having poor knowledge about malaria seemed to have poor treatment-seeking behaviour. A considerable number of respondents seek care from informal care providers and seek appropriate care late. There is a need to focus on specific targeted groups, promote awareness on the role of early diagnosis and treatment and address misconceptions about transmission of malaria.

## Abbreviations

ACT: Artemisinin based combination therapy
DMR: Department of Medical Research
DMS: Department of Medical Services
DOPH: Department of Public Health
EDAT: Early diagnosis and appropriate treatment
EDTM: Early Diagnosis and Treatment of Malaria
MOHS: Ministry of Health and Sports
NMCP: National Malaria Control Program
PPS: The probability proportionate to size
RAI: Regional Artemisinin Resistance Initiative
STROBE: The Strengthening the Reporting of Observational Studies in Epidemiology
VHV: Village health volunteer
VMW: Voluntary malaria worker
WHO: World Health Organization
